# Dental Schools in London

**Published:** 1893-09-16

**Authors:** 


### Dental Schools in London.

It used to be thought that in order to set up as a dentist all that one needed to do was to arm oneself with a forceps and to proclaim a more or less disinterested desire to relieve the sufferings of a toothache-ridden public. It is still a sore point with those who have given time and money to become qualified dental surgeons that it is so difficult to put a check upon those who practice as dentists without any qualification whatever. In this the qualified dentist has certainly a grievance; but whether it would be wise to call in the law to remedy it is a question upon which opinions differ, and it need not be discussed here. At any rate, one may hope that the public will learn to place the quack dentist in the same category as the quack doctor, and that people will come to have a greater regard for their teeth than to incur the risks that always attend the work of the untrained, and therefore unskilled, dentist.

To become a qualified dentist is by no means child's play. The course of study prescribed by the Royal College of Surgeons for the L.D.S. is long and thorough. It is required of every candidate for the degree that he shall produce evidence of having been engaged during a period of not less than three years in acquiring a practical familiarity with the details of mechanical dentistry under the instruction of a competent practitioner; and of having been engaged during four years in the acquirement of professional knowledge subsequently to the date of his registration as a dental student. The three years' pupilage in dental mechanics may be taken before or after registration, and students are advised to complete this term before entering upon their professional course. The four years' curriculum imposes attendance at a recognized medical school and general hospital in addition to the course at dental school and hospital. In respect of a large group of subjects the requirements for the L.D.S. are the same as for the L.R.C.P., and M.R.C.S. The following subjects are common to both curricula: Chemistry, anatomy, physiology, materia medica, surgery and medicine, clinical surgery, and clinical medicine. These subjects the dental student takes along with medical students at a medical school. But in addition to these there are the subjects for which he must have recourse to the special dental school. These latter are dental anatomy, and physiology—human and comparative; dental surgery, and dental mechanics.

There are three dental schools in London. Of these only one, that at Guy's, is attached to a general hospital. It was in December 1888, that it was resolved by the Governors of Guy's Hospital that the Dental department of the hospital should be extended by the establishment of a School of Dental Surgery. A conservation room with 18 dental chairs was built within the grounds, and with this room and two extraction rooms in the general out-patient department the school was opened in October, 1889, with six students. The new departure was speedily justified in the large number of dental students who came to Guy's. So rapid was the growth of the school that it became necessary to erect a new building. Upon this the governors of the hospital resolved in December, 1890; and as to the manner in which the resolution has been carried out all who have visited the dental school at Guy's, opened some weeks ago, can testify. Buildings better fitted to serve their purpose it would be difficult to imagine. They comprise a conservation room capable of containing 50 operating chairs, a large laboratory, and abundant waiting-room accommodation. The conservation room is fitted up with all the appliances which latter-day skill in dentistry has brought into existence. In the laboratory the student is enabled to pursue the study of various branches of the dentist's art under the most favourable circumstances. It is not to be denied that Guy's possesses special attractions for the dental student. Not only is an admirable training provided in all the subjects of the dental course, but the very fact that the school is attached to a general hospital is an advantage in that the student has special opportunities of realising the relation of diseases of the teeth to other diseases to which flesh is heir. The tendency to regard the welfare of the teeth as the be-all and end-all of human existence is counteracted. Moreover, it was the hope of the founders of the school that a greater number of dental students would be induced to become qualified surgeons, and that thus the position of dental surgery would be raised. Undoubtedly the close association of dental school and medical school is calculated to bring about this desirable result. At any rate, the dental school at Guy's has had unexampled success. The school was opened in 1889 with six students. Last session the number of students was 70.

At the two other dental schools, the National Dental in Great Portland Street, and the Dental School in Leicester Square, the practice is for the student to take his general course at one of the neighbouring general hospitals. Both schools are so situated as to allow of this being done. There are several general hospitals within a short distance of Leicester Square and Great Portland Street.

The National Dental School is about to remove from the buildings in which it has been housed since 1877 to a fine new hospital at the corner of Devonshire Street and Great Portland Street. The buildings that are to be abandoned were never well suited for the puposes of a dental hospital, and for some time they have been too small. For the new hospital, as well as for the site on which it is erected, the public is indebted to the great generosity of the Dowager Lady Howard de Walden. No expense has been spared to make it a model dental hospital, and it need not be doubted that when completed, as it will be in a few days, it will be found to be everything that patient and student can desire. School and hospital are well officered, and the course of instruction provided is very complete. It is worthy of note that the National Dental College is the ouly dental school to which ladies are admitted. Here ladies are prepared for the examination of the Edinburgh College of Surgeons, which is the only examining body which confers the dental diploma upon women.

Although the Dental Hospital in Leicester Square has been frequently enlarged, the tax upon space has been so great that there is a serious intention of making further enlargement, if not of building a new hospital. Our visit to this school was paid in the forenoon, and one could easily see that patients are numerous enough to make the opportunities of the student in the way of practical work ample. Conservation room, extraction room and waiting rooms were well filled. There is sufficient class room and laboratory accommodation. In the museum human and comparative dental anatomy, as well as dental pathology, are well represented. In the basement is the workshop, where the making of artificial teeth and of the various appliances of dental surgery is practiced under the superintendence of a skilled mechanic. A large staff of demonstrators give instruction in the several forms of filling-in, extraction, in the treatment of the irregularities, and the fitting-in of artificial teeth.

There are several dental schools in the provinces. Mason College, Birmingham; Owens College, Manchester; and University College, Liverpool, have each dental departments. There is a dental hospital and school in Edinburgh, and St. Mungo's College, Glasgow, receives dental students.

